# Temperature-dependent potential for the molecular dynamics of the superionic conductor β-PbF2

**DOI:** 10.1016/j.heliyon.2022.e09026

**Published:** 2022-02-26

**Authors:** J.D. López, J.E. Diosa, G. García, E. Mosquera, H. Correa

**Affiliations:** a Grupo de Transiciones de Fase y Materiales Funcionales, Departamento de Física, Universidad del Valle, Santiago de Cali, Colombia; b Centro de Excelencia en Nuevos Materiales, Universidad del Valle, Santiago de Cali, Colombia; c Facultad de Física, Pontificia Universidad Católica de Chile, Santiago de Chile, Chile; d Instituto Interdisciplinario de las Ciencias, Universidad del Quindío, Armenia, Colombia

**Keywords:** Superionic conductor, Lead fluoride (PbF_2_), Molecular dynamics (MD)

## Abstract

Molecular dynamic (MD) calculations were performed to investigate the thermodynamic and structural properties of lead fluoride (PbF_2_) by using a proposed inter-ionic temperature-dependent potential. This potential allows calculating with high precision the linear thermal expansivity and the lattice parameter as a temperature function. In addition, the potential can be represented as a sum of two contributions, a temperature-independent potential added to another temperature-dependent potential, considered last as a correction justified by the one-dimensional Newtonian quantum equation. Two fitting regions were considered, the first region from 300 to 700 K and the other one from 700 to 900 K. These regions arise naturally due to the smooth and continuous transition that PbF_2_ undergoes until it reaches the superionic state and, allows us to model with high precision the anomaly in the dependence of the lattice parameter with the temperature of this material, a feature that until now under the molecular dynamic method has not been studied. These results are all in good agreement with the experimental measurements.

## 1 Introduction

Computational simulations applied to ionic systems are a powerful tool that allows us to study and predict their behavior when they are subjected to external stimuli, which in most cases cannot be achieved in the laboratory. Particularly, molecular dynamics (MD) is a classical method that bases its algorithm on the resolution of Newtonian motion equations [1], which allows the study of large ionic systems at a low computational cost. The quantum effects of each ion can be disregarded (i.e., electronic structure) because the choice of an effective interaction potential, acts manifesting the characteristics as a global average (see Eq. (1)). Therefore, these potentials are derived from quantum or empirical considerations. For this reason, a right choice in the mathematical form of the potentials is crucial to guarantee a behavior like the real one. In molecular dynamics (MD), these interaction potentials are adjusted to describe the actual behavior of physical systems employing constants, and it depends explicitly on the distance between the ions or atoms. However, if a temperature gradient is applied to the system, the effect of the temperature only manifests itself as a contribution to the kinetic energy of each atom [2]. Nevertheless, it is expected that the temperature slightly modifies the interactions between the ions. Due to this, it is necessary to include temperature-dependent corrections to the interaction potential. Currently, few studies employ a methodology that reveals a dependence on interaction potentials with temperature variation. Subramaniyan and Sun [3] developed an alternative to classical molecular dynamics called engineering molecular mechanics (EMM), which allows simulating physical properties in materials at high temperatures (T > 0 K). They employed the coefficient of thermal expansion (CTE), which is a well-studied property, converting the interaction potential into a temperature-dependent potential by allowing the adjustment constants to depend on the CTE. Although the authors manage to optimize the computation time, this method is based on the molecular statics (MS) and not on the MD. Instead, Nasehzadeh et al, [4] reevaluated the adjustment parameters of the Lennard-Jones potential and improve the calculation of the thermodynamic properties of liquids. They used the Chandler-Week-Andersen perturbation theory, which provides a precise analytical expression and allows determining that both *σ* and *ε* can be expressed as a linear function of temperature. Other works that respond to more particular problems and that consider this type of dependency can be found in Refs. [5, 6, 7].

On the other hand, the study of ionic conductors through the use of MD is a current scientific topic, since there is still no precise understanding of the mechanisms involved in the ionic conductivity of the superionic state [8, 9, 10, 11]. One of the most relevant superionic conductors with fluorite structure is β-PbF_2_, which has a structure described as an FCC array of lead cations (Pb^2+^), where all the tetrahedrally coordinated interstitial sites are filled with fluorine anions (F^–^), being F^–^ the mobile species. The lattice parameter, their density and specific heat capacity are 0.593 nm, 7.750 g/cm^3^ and 301 J Kg^−1^ K^−1^, respectively. The phase transition to the superionic state, undergone by this compound, is accompanied by a λ-type peak in the specific heat, no reorganization of the crystalline frame structure, and a smooth and continuous change in conductivity as the temperature increases [12]. Likewise, this material has an anomaly in its thermal expansion when it reaches the transition temperature towards the superionic state (711 K), and there are still no studies that can describe this behavior in the lattice parameter by means of MD. In this work, the theory and methodology proposed by Nagornov and Katz [7] is used, this shows a new approach to the formulation of interaction potentials in MD by means of semiclassical considerations it is possible to express the interaction potential as a sum of an independent part of the temperature and temperature-dependent as a perturbation, as is shown in Section 3.1. Its main application is those physical phenomena, where the variation of the temperature takes a crucial role. This work validates a new methodology for obtaining this type of potentials, using crystalline structural variations with temperature for an ionic crystal-like PbF_2_.

## 2 Methods

The simulations were made using the DL_POLY Classic 1.9 package developed by Smith *et al.* [13] at the Daresbury Laboratory. The initial simulation was performed in a cubic simulation box of long size 23.720 Å, made with VESTA® software, that corresponds to a 4 × 4 × 4 supercell, using 768 atoms, 256 lead cations and 512 fluoride anions. Artificial periodic boundary conditions have been used to reproduce the bulk properties. The initial system was equilibrated at environmental conditions (300 K, 1 atm) during 500000 steps of 2 fs that allows to compare with experimental measurements. An Intel® Core™ i5-7200U with turbo boost up to 3.1 GHz and 16 GB RAM PC was employed to perform the simulations.

The calculations were carried out using integration times of 2 fs and cutoff radii of 10 Å employing the Verlet method. The sum of Ewald was used to compute the Coulomb term of the potentials presented in Eq. (4). To perform the calculations of the thermodynamic properties, the system was configured under the canonical NVT ensemble, in which the temperature is increased, and subsequently, the properties in the NpT assembly are registered in order to maintained constant temperature and pressure, using an artificial Nosé – Hoover thermostat and barostat respectively which allows making a comparison between the simulated data with experimental data, during 100000 steps of 2 fs, were used to let the system stabilized until the recording process.

## 3 Results and discussion

### 3.1 Construction of a temperature-dependent interatomic potential

#### 3.1.1 Semi-classical considerations

Following a semi-classical treatment from the Ehrenfest theorems [14], it is possible to derive the one-dimensional (1D) Newtonian quantum equation:

(1) $$\mu \frac{\partial^{2}\bar{x}}{\partial t}=\frac{-\partial U_{eff}\left(\bar{x},T\right)}{\partial \bar{x}}$$

where μ is the atom wave packet mass, $\bar{x}$ is the wave packet coordinate and $U_{eff}\left(\bar{x},T\right)$ is an effective potential dependent on temperature, where

(2) $$\frac{\partial U_{eff}\left(\bar{x},T\right)}{\partial \bar{x}}=\frac{\partial U\left(\bar{x}\right)}{\partial \bar{x}}+\frac{1}{2!}\frac{\partial^{3}U\left(\bar{x}\right)}{\partial {\bar{x}}^{3}}\frac{1}{e^{\gamma }N}\underset{s}{\sum }\Delta {\bar{x_{s}}}^{2}g_{s}exp\left(\frac{\varepsilon_{s}}{kT}\right)+\ldots$$

In Eq. (2) N is the number of atoms in the assembly, $e^{\gamma }$ is a normalization coefficient, $g_{s}$ is the number of single-particle states and $\varepsilon_{s}$ is the energy of the atom in the s state [7]. The effective potential is a sum of two terms, a potential that depends on the temperature and another independent, such that the temperature-dependent part varies small amounts respect to U(x) like:

(3) $$U_{eff}\left(x,T\right)\approx U\left(x\right)+\delta U\left(x,T\right)$$

Thus, the problem is reduced to find the temperature-dependence of the interionic interaction potentials satisfying the Eq. (3).

#### 3.1.2 The Buckingham potential in the β-PbF_2_

The crystalline ionic solids with fluorite-like structure, such as PbF_2_, CaF_2_, BaF_2_, and so on, are often well described by the potential U(x) that involves the Born-Mayer-Huggins form:

(4) $$U\left(r_{ij}\right)=A_{ij}exp\left(\frac{-r_{ij}}{\rho_{ij}}\right)-\frac{C_{ij}}{r_{ij}^{6}}+\frac{q_{i}q_{j}}{r_{ij}}$$

The first two terms on the right side of Eq. (4) are known as Buckingham's potential, being $r_{ij}$ the distance between the *i*-th and *j*-th ions. The exponential term represents electronic overlap (energy repulsion), the second term is proportional to $-1/r_{ij}^{6}$ being the dispersion term, and the last term is the potential due to the Coulomb interactions.

For the β-PbF_2_, the adjustment parameters most frequently used in the literature for independent-temperature potentials are those proposed by Walker et al. [15], as shown in Table 1.

**Table 1:** Adjustment constants of the potentials that describe the β-PbF_2_ by MD, as proposed by Walker et al. [15].

Atomic pairs	*A*_*ij*_/eV	$\rho_{ij}$/Å	*C*_*ij*_/eV Å^6^
Pb – Pb	0.0	0.0	0.0
Pb – F	122.7	0.516	0.0
F – F	10255	0.225	107.3

In this work, the parameter $\rho_{Pb-F}$ was considered as a function of the temperature, with a linear relation:

(5) $$\rho =\rho_{0}\left(1+\eta T\right)$$

In a similar way to Lennard-Jones coefficients [4] and the pre-exponential factor of the compact representation of Buckingham's potential [7]. By replacing the Eq. (5) in $U\left(r_{Pb-F}\right)$ given by Eq. (4) obtains:

(6) $$U\left(r_{ij},T\right)=A_{ij}exp\left[\frac{-r_{ij}}{\rho_{0}\left(1+\eta T\right)}\right]\approx A_{ij}exp\left[\frac{-1}{\rho_{0}}\left(1-\eta T\right)r_{ij}\right]$$

The result of the right side (Eq. (6)) is obtained by expanding the argument of the exponential in a series of power of temperature and using the fact that it is possible to rewrite this equation, therefore, the temperature-dependent part is:

(7) $$A_{ij}exp\left[\frac{-r_{ij}}{\rho_{0}}\right]exp\left[\frac{\eta r_{ij}}{\rho_{0}}T\right]\approx A_{ij}exp\left[\frac{-r_{ij}}{\rho_{0}}\right]\left(1+\frac{\eta r_{ij}}{\rho_{0}}T\right)$$

However, Eq. (7) shows that $U\left(r_{ij},T\right)\approx U\left(r_{ij}\right)\pm \delta U\left(r_{ij},T\right)$, which is the condition imposed in Eq. (3), that the potential must satisfy.

#### 3.1.3 Determination of the temperature-dependent parameter

**Figure 1:**
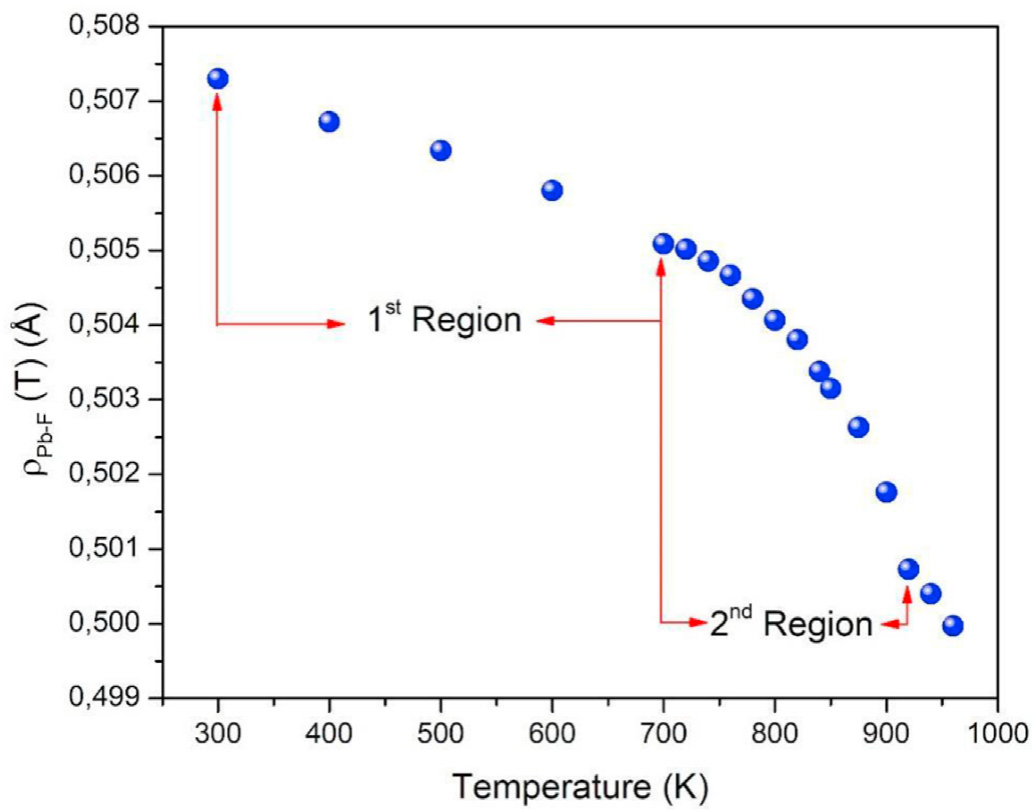
Dependence of the fitting parameter $\rho_{Pb-F}$ with temperature.

In order to determinate the temperature dependence of the $\rho_{Pb-F}$ parameter, this is slightly modified from 0.490 to 0.520 eV for 300, 400, 500 and 600 K, and modified from 0.490 to 0.510 eV for 700–900 K [16], performing a new simulation set with the previous conditions. For each temperature, the $\rho_{Pb-F}$ is obtained to adjust the experimental lattice parameter, from the $\rho_{Pb-F}$ versus lattice parameter plot. Finally, the $\rho_{Pb-F}$ values versus the temperature are plot as shown in Figure 1, where three regions can be seen: the first at low temperatures, that is, before the superionic state, a second region up to 920 K and a third region for temperatures above this value. The calculations in the third region were not considered, due to inconsistencies in the calculations of the transport properties.

It is interesting to note how linearity is lost when passing from the non-superionic to the superionic region, which naturally justifies the need for two adjustment regions, one linear and one of a higher order > O(1). To find the best numerical adjustment, the continuity of the function ρ(T) is waived, however, the discontinuity between both sections is so small that it can be neglected for numerical purposes (≈2.275 × 10^−4^ Å). The dependence of the fitting parameter with the temperature was found in Ref [16] as:

(8) $$\rho_{Pb-F}\left(T\right)=\left\{ \begin{array}{l} 0.5089Å-3.34\times 10^{-6}TÅ/K,if300K\leq T\leq 700K\\ 0.4654Å+1.1360\times 10^{-4}TÅ/K-8.1603\times 10^{-8}T^{2}Å/{\text{K}}^{2},if700K<T\leq 920K \end{array}\right.$$

**Figure 2:**
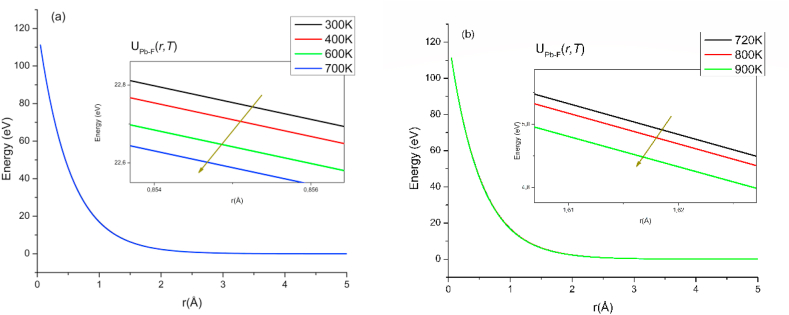
Component $U_{Pb-F}$ of the potential for different temperatures, from (a) region 1 and (b) region 2.

It is found that as the temperature increases, the parameter $\rho_{Pb-F}$ decreases and therefore the potential suffers a decrease, causing the fluoride and lead ions to experience a lower repulsion force with each other. While it might be expected that the repulsion between the ions would be greater because the crystal expands with temperature, this behavior arises as compensation for the classical thermal energy that is imparted to each ion. Likewise, a decrease in potential energy allows fluorine ions to move more easily between available sites in the lattice. Figure 2 shows the behavior of the potential of the Pb–F interaction, in the region of low (Figure 2a) and high (Figure 2b) temperatures. It is inferred that the proposed modification affects the potential in the order of tenths of eV by varying the thermal bath temperature by ± 100 K, corroborating that the Eq. (8) agrees with the Eq. (3).

### 3.2 Validation of interatomic potential

#### 3.2.1 Structural stability

**Figure 3:**
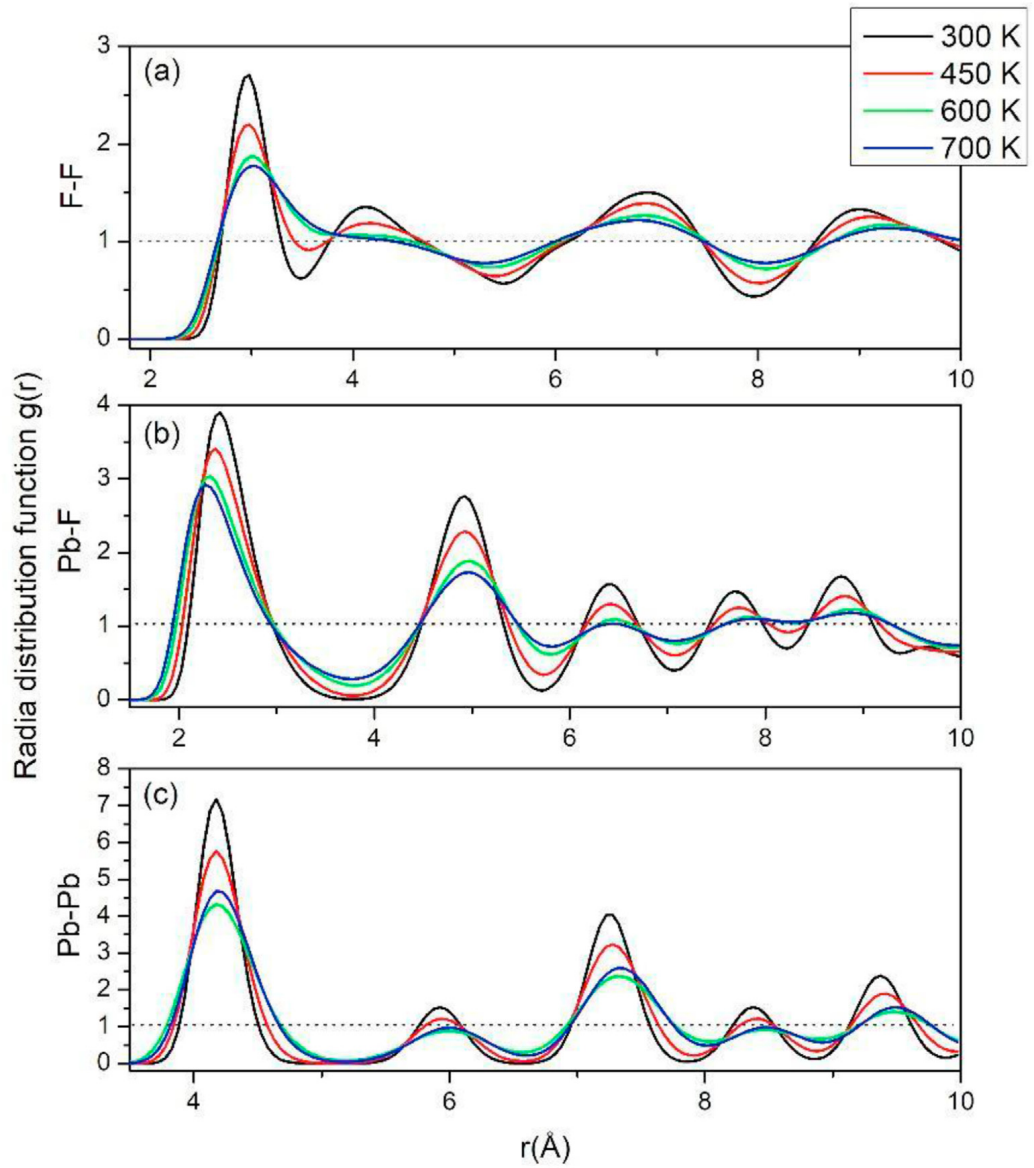
Function of radial distribution of β-PbF_2_ at different temperatures, for (a) F–F, (b) Pb – F, (c) Pb–Pb.

**Figure 4:**
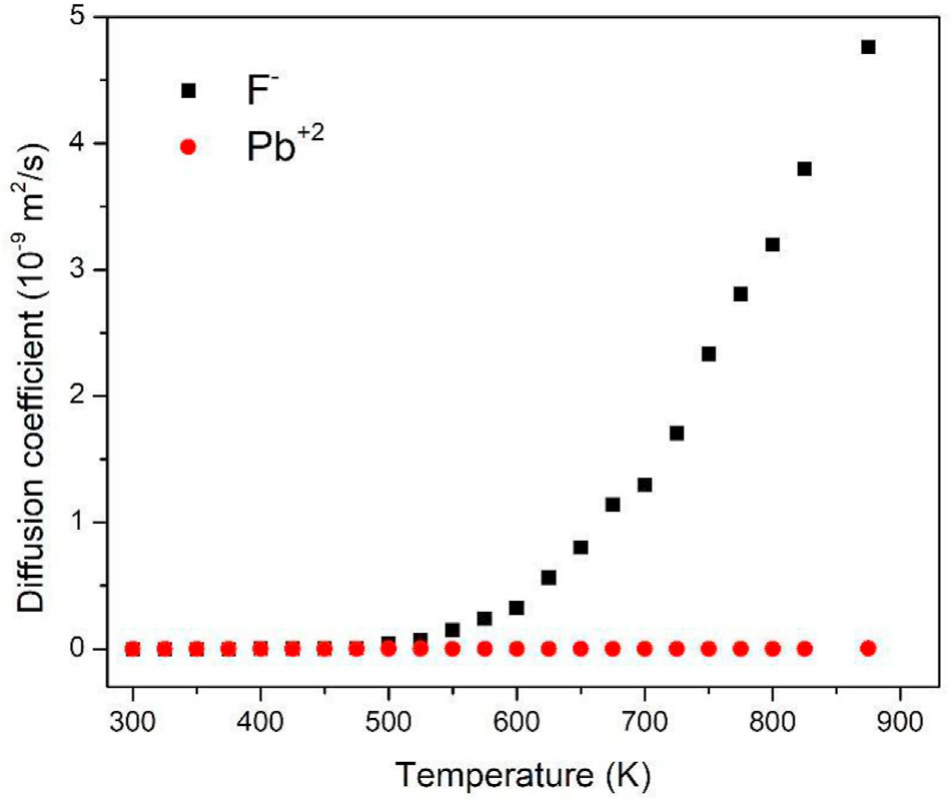
Coefficient of diffusion against temperature for ionic fluorine and lead spices.

To verify that the proposed potential does not lead to the study material undergoing a structural phase transition, or an undesired disorder in any of its sublattice with the rise of the temperature, the radial distribution functions *g*(r) is calculated. This function allows knowing how the ions are distributed from the atoms of the material with respect to a type of ion in a spherical shell (refer to Figure 3). The *g*(r) has a first well-defined maximum attributed to the first neighbors, and as the temperature increases, it loses its shape, decreasing their intensity and increasing their width, because the ions experience greater mobility as the temperature increases of the system. However, unlike lead ions (Figure 3c), the *g*(r) of fluorine ions (Figure 3a) changes to a radial distribution function, similar in shape to liquid materials. This behavior partly explains the superionic conductivity in this kind of material, where the sublattice of the mobile ions, has a quasi-liquid behavior due to its high mobility and diffusion, which can be corroborated in Figure 4, in which the ions of lead remain in their network sites, while those of fluorine begin to diffuse gradually through the crystal as the temperature increases. From Figure 3b, a mean distance of 2.42 Å between the lead and fluorine ions at room temperature was calculated. This distance turned out to be slightly greater than that reported by Silva et al. [17], using temperature-independent potentials, but lower than that experimentally calculated (2.57 Å). Figure 3b, show how the coordination distance decreases as the temperature increases.

#### 3.2.2 Thermal expansion coefficient

**Figure 5:**
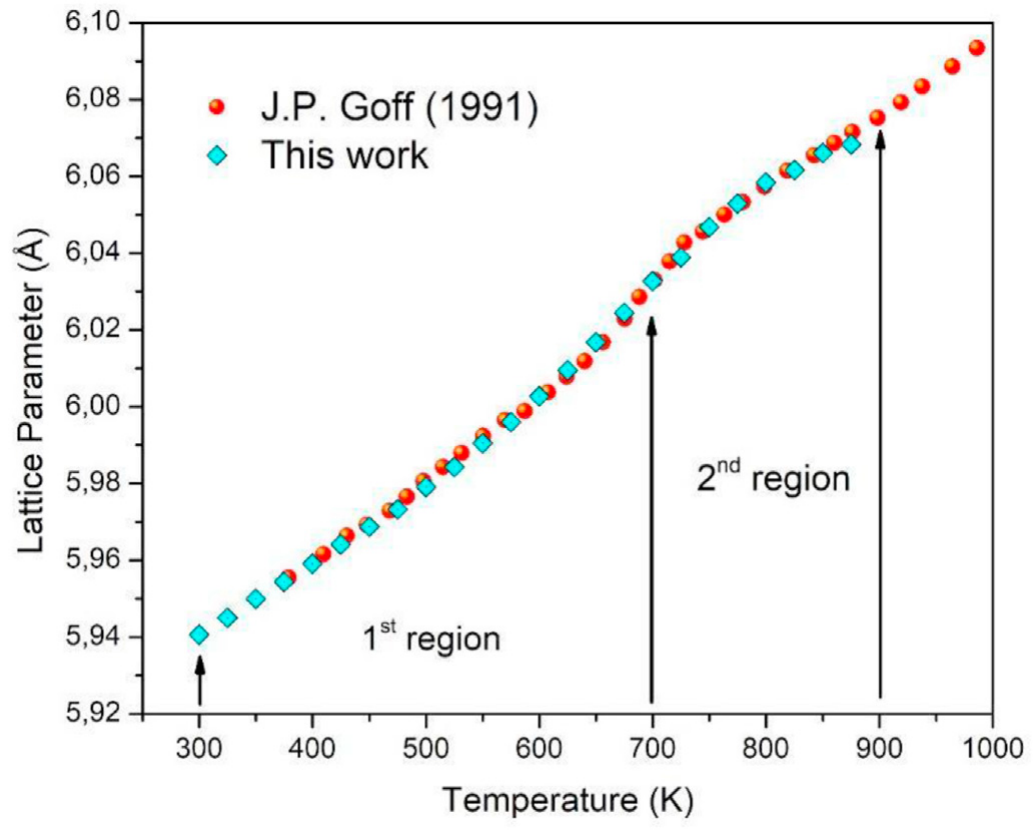
Dependence of the lattice parameter with temperature.

Figure 5, shows the variation of the lattice parameter when the temperature of the system, is increased and compared with the results obtained experimentally by Goff et al. [18], (in red circles), against those calculated in this paper (in cyan diamonds); when using the modification proposed in Eq. (8). A high coincidence with that reported experimentally is shown, even fitting to the anomaly present near 700 K, so the choice of two regions shows to be an appropriate process.

Once the dependency of the lattice parameter $a_{0}\left(T\right)$, with the temperature, is found, it is possible to find the linear thermal expansion coefficient α (see Eq. (9)) as [19]:

(9) $$\alpha =\frac{da_{0}}{dT}\frac{1}{a_{0}\left(293K\right)}$$

**Figure 6:**
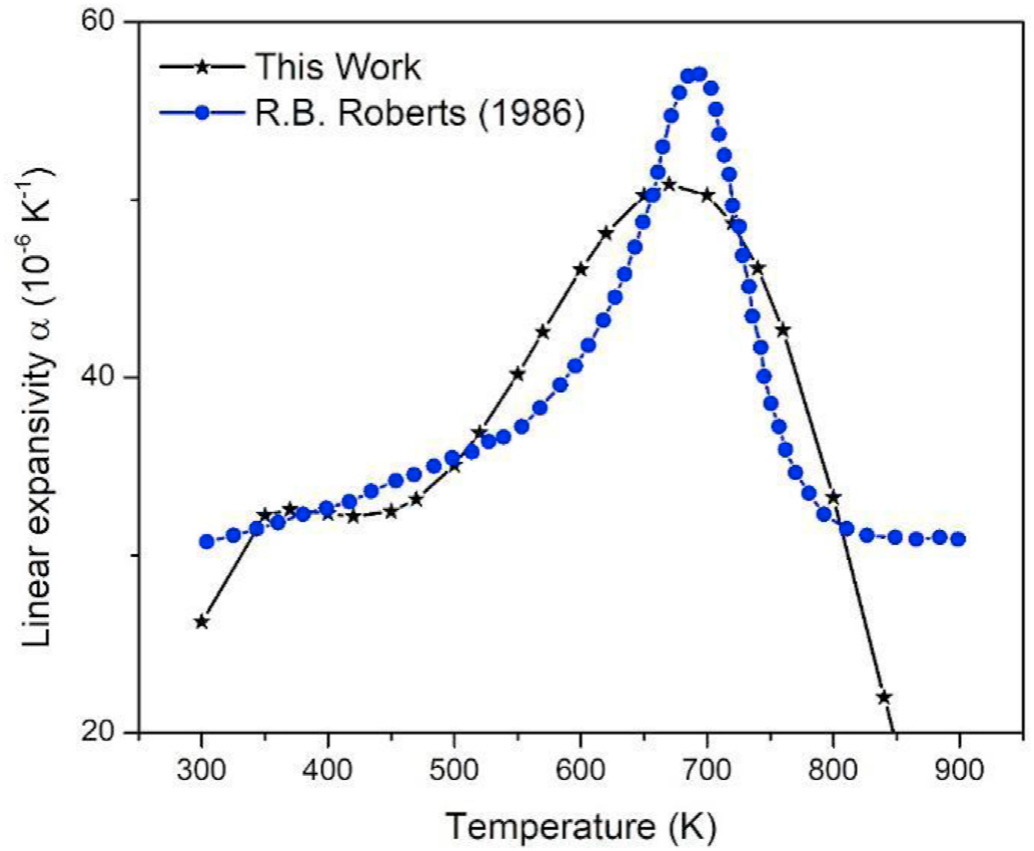
Comparison of linear thermal expansion coefficient with temperature, in blue circles, found experimentally by Roberts and White [20] and in black stars, as calculated in this work.

A sixth-grade polynomial fit for the data obtained in this work was done, and its gradient was calculated (see Figure 6) where it is compared with experimental data of the linear thermal expansion coefficient obtained by Roberts and White [20]. It is interesting to note that the calculated curve has a maximum close to 670 K while the experimental curve around 690 K. However, both have the same line shape and order of magnitude, with an error slightly lower than 30% in area, in the range of 350–800 K. Beyond 800 K, the data deviates from experimental behavior. Also, it is the first time that this type of graph has been made by MD calculations for β-PbF_2_.

#### 3.2.3 Heat capacity

The heat capacity ($C_{p}$) of any material is defined as the amount of heat per unit of mass required so that its temperature raises 1 K [21]. For the molecular dynamic case, $C_{p}$ is calculate by:

(10) $$C_{p}=\frac{1}{n}{\left(\frac{\partial H}{\partial T}\right)}_{NpT}$$

**Figure 7:**
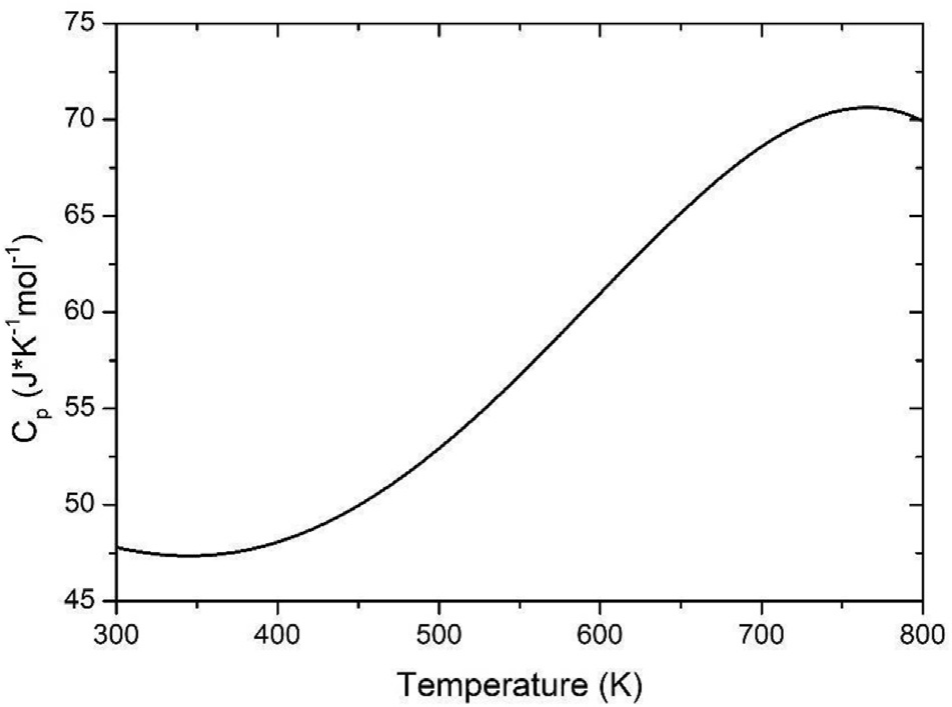
Heat capacity as a function of temperature.

Being *n* the number of moles, *H* the enthalpy computed in the NpT ensemble of the system, and T is the crystal temperature. The $C_{p}$ curve calculated from Eq. (10), from 300 K to 800 K is plotted in Figure 7. The $C_{p}$ increases in a wide region around 700 K, near the superionic transition, predicting a phase transformation to the superionic state due to an increase in internal energy, related to the mobility of mobile ions. In previous study [11], the $C_{p}$ values employing only the Walker's potential showing a higher value (80.49 J K^−1^ mol^−1^) than the experimental one (76.81 ± 0.51 J K^−1^ mol^−1^ at 400 K) and above 700 K a chaotic behavior arise, without exhibiting a phase transition. In this work, a lower value $C_{p}$, than the experimental is calculated (52.3 J mol^−1^ K^−1^ at 500 K against 79.87 J mol^−1^ K^−1^ measured by Volodich et al., [22]), and a phase transition is predicted. The difference between the values (experimental and theoretical) is the non-existence of a nominal value in the literature for temperatures above room temperature. However, a similarity in the shape of the lines can be appreciated. From temperatures above 800 K, a difficulty arises in fitting the enthalpy data.

## 4 Conclusions

In summary, the potential proposed by Walker et al. [15], was modified, to describe the structural properties of β-PbF_2_ as a temperature function, by making this equation parametrically dependent on temperature. The fitting constant $\rho_{Pb-F}$ was replaced by a linear function $\rho_{Pb-F}\left(T\right)$. This parameter was fitted with experimental data of the lattice parameter versus temperature. It was found that two adjustment regions naturally emerged, the first one from 300 to 700 K and another from 700 to 900 K. The first is characterized by having a linear behavior, while the second exhibits a second-order polynomial form. From these functions, calculations by molecular dynamics were made, and it is possible to conclude that there exists a high coincidence between the data of the evolution of the lattice parameter with the temperature and those calculated with our proposed potential, it even fits the anomaly present in the transition temperature towards the superionic state. From the g(r) function obtained, it is possible to conclude that the proposed potential does not involve a structural phase transition, and the ions distance is almost equal to the experimental one. The $C_{p}$ curve is firstly for this material calculated from 300 to 800 K, exhibiting a growing line shape, until a maximum; then, around it, a phase transition towards the superionic state is predicted.

## Declarations

### Author contribution statement

López J.D.: Conceived and designed the experiments; Performed the experiments; Analyzed and interpreted the data; Contributed materials, analysis tools or data; Wrote the paper.

Diosa J.E., H. Correa: Conceived and designed the experiments; Analyzed and interpreted the data.

García G.: Conceived and designed the experiments; Contributed materials, analysis tools or data.

Mosquera E.: Analyzed and interpreted the data; Wrote the paper.

### Funding statement

This work was supported by Ministerio de Ciencia Tecnología e Innovación, Colombia(Grant no. C.I. 1165).

### Data availability statement

Data associated with this study has been deposited at Data in Brief - Volume 28, February 2020, 104865 (https://doi.org/10.1016/j.dib.2019.104865).

### Declaration of interests statement

The authors declare no conflict of interest.

### Additional information

No additional information is available for this paper.
